# HEARING LOSS AMONG OLDER ADULTS IN THE NATIONAL HEALTH AGING TRENDS STUDY

**DOI:** 10.1093/geroni/igad104.0763

**Published:** 2023-12-21

**Authors:** Nicholas Reed

**Affiliations:** Johns Hopkins Bloomberg School of Public Health, Baltimore, Maryland, United States

## Abstract

Hearing loss is an important condition that affects healthy aging. However, nationally representative estimates of hearing loss among older adults are limited due to differences in measures and categorization of hearing as well as limited sampling of older adults. The aims of this presentation are to review the meaning of different hearing measures and categorization and to estimate the prevalence of hearing loss by age and demographic covariates of adults over 70 years. The National Health and Aging Trends Study (NHATS) is a large nationally representative panel study of Medicare beneficiaries aged 65 and older with robust oversampling of the oldest old adults that added pure-tone audiometry, the current gold standard for quantifying hearing loss to the 2021 NHATS round 11 protocol. Cross-sectional (n=2620) weighted prevalence estimates by age, sex, race/ethnicity, education, and income were applied to corresponding United States Census Bureau estimates. Using traditional definitions of hearing loss, an estimated 67.9% (22.4 million) of adults over 70 years have at least some degree of hearing loss (37.4% mild, 25.8% moderate, and 4.6% severe). Prevalence is higher among White, male, lower-income, and lower education attainment subpopulations and increases with age such that 97.6% of adults 90 years and older have hearing loss. Application of new World Health Organization categories of hearing loss resulted in an increase in prevalence from 67.9% to 82.7% of adults over 70 years. The near ubiquitous nature of hearing loss in this population raises questions about identifying actionable cut points in future research.

